# Utilizing the lid of SL sheath packaging for a water‐seal catheter insertion technique

**DOI:** 10.1002/joa3.70021

**Published:** 2025-02-07

**Authors:** Tatsuya Hayashi, Shingo Yamamoto, Jumpei Ohashi, Hideo Fujita

**Affiliations:** ^1^ Division of Cardiovascular Medicine, Saitama Medical Center Jichi Medical University Saitama Japan

**Keywords:** catheter ablation, sheath, water seal

## Abstract

In the era of cryoablation and pulsed‐field ablation, where large sheaths are commonly used, preventing air embolism is crucial. The lid from SL sheath packaging can be repurposed as a water‐seal device for catheter insertion, thereby eliminating the need for additional equipment.

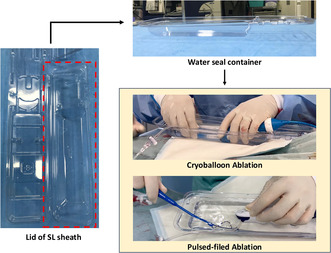

In the era of cryoablation and pulsed‐field ablation (PFA), which are increasingly performed alongside radiofrequency (RF) ablation, the sheath diameter used for left atrial access has grown larger.^1^, ^2^ This change has heightened concerns about the risk of air embolism caused by residual air in the sheath.^3^ Balloon massage and re‐flushing before catheter insertion are fundamental techniques for preventing air embolism. Additionally, submerging the sheath in a water‐filled container and inserting the catheter under water has proven to effectively eliminate air intrusion during flushing, making this approach highly effective. In Japan, Airtray® (Nisso Corporation, Shizuoka, Japan) is available as a dedicated container for this purpose, costing approximately ¥2700 per unit. However, the availability of this product is limited to certain countries or regions. As an alternative, the lid of the packaging for the SL sheath (Swartz™ Braided Transseptal Guiding Introducers SL Series, Abbott, Minneapolis, MN, USA), commonly used during catheter ablation, can be repurposed to create a water seal. The lid's larger upper section has a sufficiently deep base and is attached at only two points, which can be easily separated using scissors or similar tools (Figure 1). This repurposed lid serves as a practical container for the water seal technique, especially during cryoballoon ablation (Figure 2, Video S1) or PFA procedures (Figure 3, Video S2). The container has a depth of 35 mm, allowing the posterior end of the sheath to be submerged. To achieve full submersion, the sheath may need to be slightly bent. However, with the water‐seal technique, the tip of the sheath remains stable within the left atrium, and the sheath is quickly straightened once the catheter is partially inserted. This minimizes any potential impact on the left atrium, ensuring both safety and efficacy. Notably, this method has been employed in approximately 500 cases at our institution without any complications attributable to the technique. For optimal results, it is crucial to insert the catheter as vertically as possible into the slightly bent sheath within the container. By repurposing packaging materials that would otherwise be discarded, this method eliminates the need for additional equipment, offering a practical, sustainable, and cost‐effective solution for creating a water seal.

**FIGURE 1:**
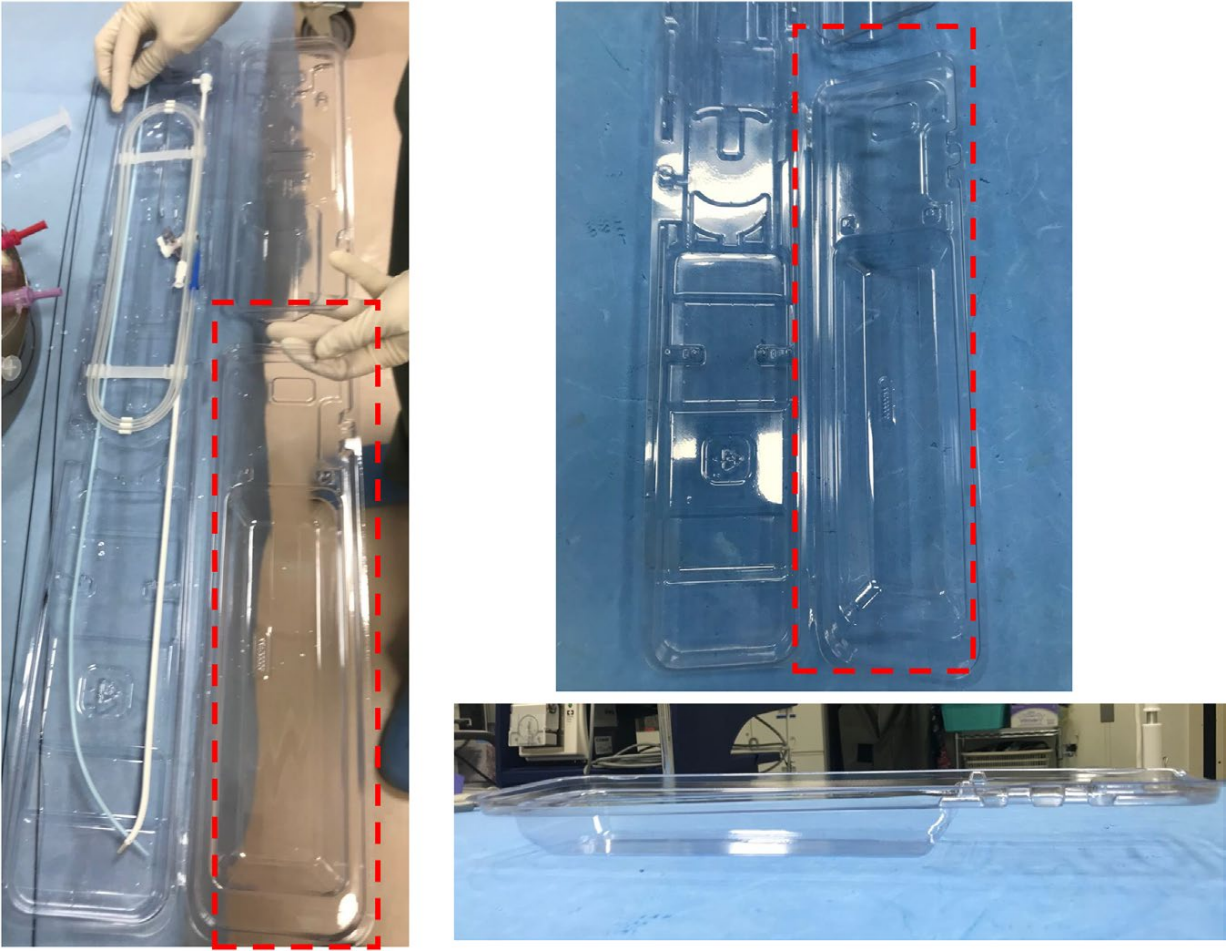
The red‐marked areas on the packaging indicate sections with a deep base, which are attached at only two points. These attachment points can be easily separated using scissors or similar tools, allowing for quick and effortless preparation for reuse. A side view of the SL sheath lid is also displayed.

**FIGURE 2:**
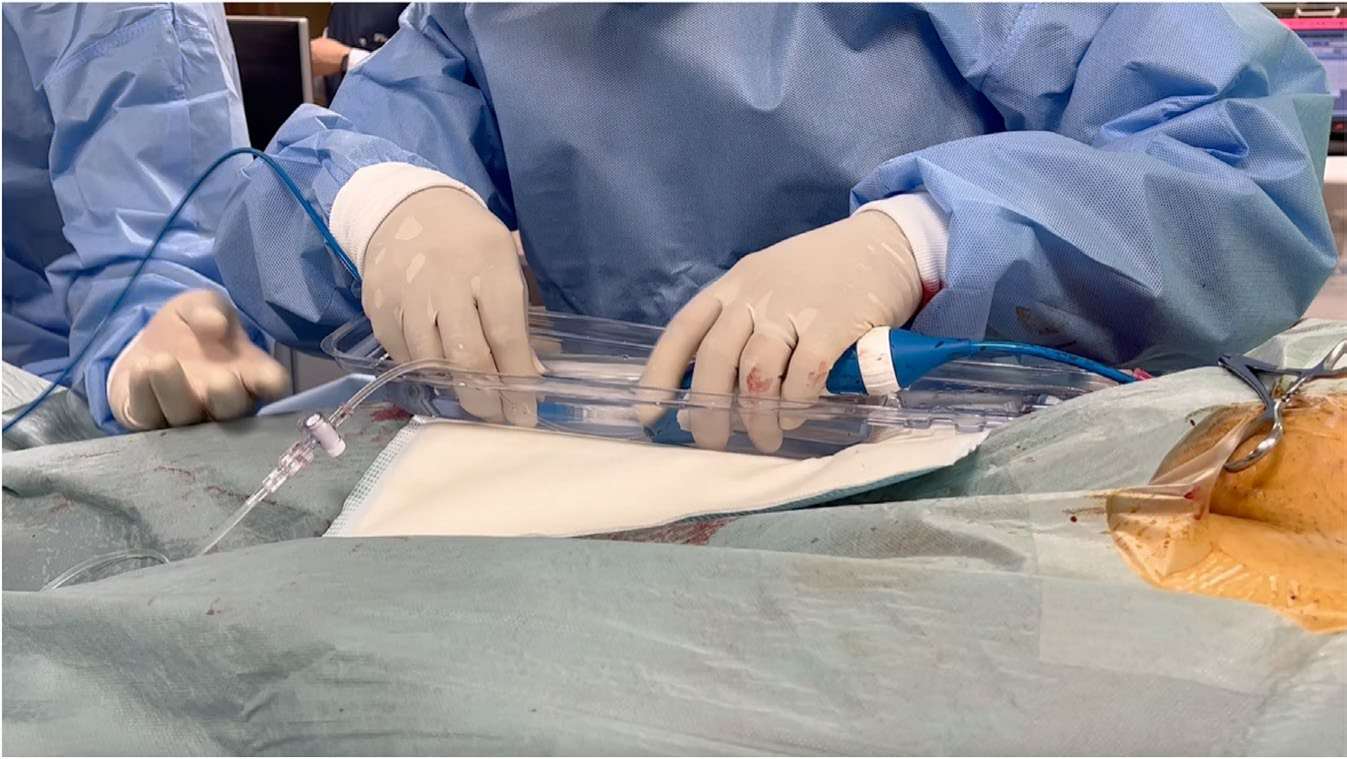
The SL sheath lid is utilized as a water‐sealing container during cryoballoon ablation (Arctic Front Advance Medtronic, Minneapolis, MN, USA).

**FIGURE 3:**
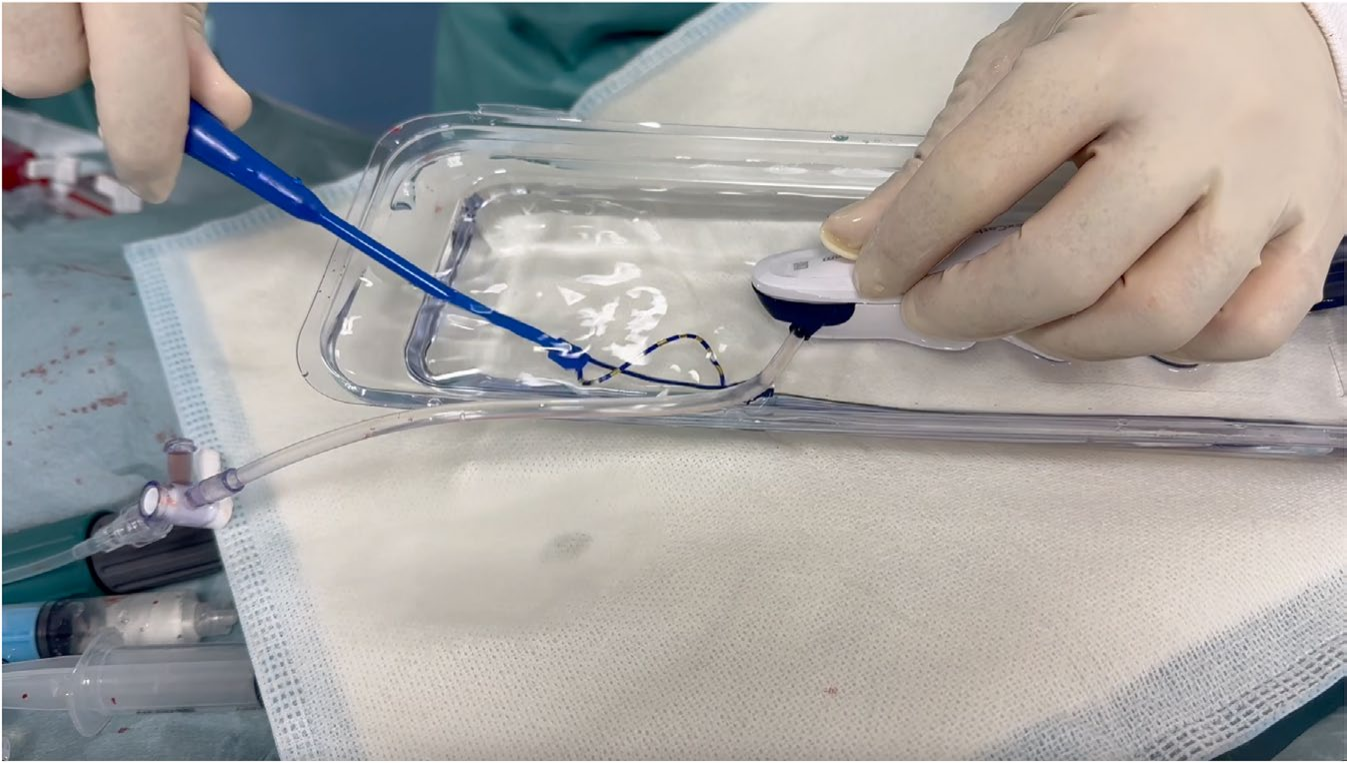
The SL sheath lid is utilized as a water‐sealing container during pulsed‐field ablation (PulseSelect Catheter Medtronic, Minneapolis, MN, USA).

## CONFLICT OF INTEREST STATEMENT

Authors declare no conflict of interests for this article.

## Supporting information


Video S1.

Video S2.

## Data Availability

Available upon reasonable request.
